# Breaking barriers: A qualitative study exploring the social and cultural factors that influence HIV awareness and uptake of HIV prevention among women of African and Caribbean heritage in England

**DOI:** 10.1177/17455057251385798

**Published:** 2025-10-22

**Authors:** Nicola Jones, Sarah Bekaert, Dianne Regisford, Nicole Jameelah Shodunke

**Affiliations:** 1 Terrence Higgins Trust, London, UK; 2 Oxford School of Nursing and Midwifery, Oxford Brookes University, UK; 3 African Families in the UK, Oxford, UK; 4 Transition Lighthouse, Oxford, UK

**Keywords:** HIV, AIDS, prevention, women, community, qualitative, African, Caribbean, participation

## Abstract

**Background:**

There have been impressive advancements made in preventing and treating HIV. However, the impacts have not been experienced equally across demographics in England. African born persons, in particular women of Black African ethnicity, continue to be disproportionately affected by HIV. HIV prevention campaigns have often overlooked social, cultural, and structural factors that shape access to HIV prevention.

**Objectives:**

This paper explores how women of African and Caribbean heritage understand HIV within their social networks, and how they perceive HIV and sexual health services. This insight is needed to develop more inclusive and effective approaches to HIV prevention.

**Design:**

This study adopted a participatory action research framework, acknowledging lived experience as a basis of knowledge.

**Methods:**

Focus groups and one-to-one interviews were used to collect qualitative data from a local community women’s group between June 2021 and November 2021. In total, 23 women of African and Caribbean heritage participated in this study. Four women opted to take part in a one-to-one interview, and 19 women participated in one of five focus groups. Data were analysed using an applied thematic analysis.

**Results:**

Three key themes emerged from the data. *Enduring fear of HIV rooted in past lived experiences:* Fear of HIV derives from traumatic lived experiences, which have shaped trust in health care services in England and limited the acceptance of information about HIV treatment, such as U=U (Undetectable = Untransmittable). *Responsibility for HIV transmission and prevention:* There was a low perception of HIV acquisition risk, shaped by personal circumstances and stigmatising beliefs. For those acknowledging they may be at risk of HIV acquisition attributed this to the behaviour of their male partners. *Motivation to change attitudes and reduce stigma:* Participating in this study provided a safe space for women to share their experiences of HIV, ask questions, and learn new information, revealing focus groups as an effective method of raising awareness and reducing stigma.

**Conclusions:**

Multiple factors shape the understanding of HIV and uptake of HIV prevention among women of African and Caribbean heritage. Local, community-specific, and participatory HIV prevention interventions are needed to understand and respond to these factors.

## Introduction

At the end of 2023, approximately 39.9 million people were living with Human Immunodeficiency Virus (HIV) globally.^1^ Although HIV has had worldwide impacts, the global experience has been diverse.^2^ In the United Kingdon (UK), gay and bisexual men were disproportionately affected by the Acquired Immunodeficiency Syndrome – AIDS crisis; in 2004, they accounted for three quarters of HIV diagnoses.^3^ Consequently, HIV prevention interventions in the UK have predominantly targeted this demographic, which has facilitated uptake in HIV prevention methods, such as pre-exposure prophylaxis (PrEP) and regular testing, resulting in declines in diagnoses.^4^

More recently, data have revealed that HIV disproportionately affects other demographics. In 2021, at the time of this research, African-born persons accounted for 42% (373 of 880) of all heterosexually acquired cases of HIV first diagnosed in England, and, notably, 61% of HIV diagnoses among heterosexual people of Black African ethnicity were at a late stage of infection. The data also indicate a gender disparity with women accounting for 63% (206 of 329) of all new heterosexually acquired HIV diagnoses made in England among people of Black African ethnicity.^4^ A report from the local council where this research was conducted revealed a similar pattern and highlighted a low uptake of full sexual health screens (those that include tests for HIV and syphilis, alongside chlamydia and gonorrhoea) among Black women.^5^ Furthermore, data indicate that women of Black African ethnicity are least likely to be offered an HIV test when accessing specialist sexual health services, indicating barriers at both individual and service level.^4^ Consequently, women of Black African ethnicity are often accessing HIV care late, and uptake of prevention methods has been modest compared with other demographics.^6–8^

To improve uptake of HIV prevention among women of Black African ethnicity, their beliefs and experiences of HIV must be understood.^9^ However, HIV awareness campaigns have often overlooked social, cultural, and structural factors that shape engagement with services, and have tended to adopt top-down approaches centred on the provision of education to change behaviour.^10,11^ Historically, the UK government ‘Don’t aid AIDS’ campaign in the 1980s, better remembered as the ‘don’t die of ignorance’ or ‘tombstone’ campaign, epitomised this through focusing on abstinence and safe sex, and relying on fear to engender behaviour change.^12^ This campaign did little to support the emotional needs for those diagnosed with HIV, leaving them scared to disclose to family and friends; a fear that persists today.^12^

Knowledge and treatment of HIV have dramatically improved over the past 40 years. In turn, national campaigns, for example, ‘It Starts with Me’ from HIV Prevention England (managed by Terrence Higgins Trust), have adopted more inclusive approaches to HIV prevention that encompass raising awareness, improvement of attitudes, and encourage the uptake of protective behaviours, for example, testing, PrEP, and treatment as prevention.^13^ However, national campaigns face the challenge of disseminating messages that resonate with diverse audiences, all of whom have different perceptions and knowledge of HIV. The ‘I Test’ campaign as part of National HIV Testing Week has made some progress in overcoming this challenge by allowing for versatile sub messages that appeal to different audiences, e.g. ‘I Test *because HIV can affect anyone’*.^14^

To build on this progress and minimise HIV associated morbidity and mortality, particularly among groups where HIV incidence and late diagnoses are higher, it is necessary to complement national campaigns with local tailored approaches. To achieve this, more insight into the factors that shape access to HIV prevention among groups most affected is needed; in relation to this project, women of African and Caribbean heritage, whose perspectives on HIV and prevention campaigns have often been overlooked.

The introduction has set out the general historical and contemporaneous context for HIV prevention, within which women of African and Caribbean heritage assess their risk and negotiate service provision. Presentation of key social and cultural factors typically ascribed to Black African and Caribbean women have been purposefully avoided in the introduction. The authors have attempted to avoid prescribing explanations for low engagement with sexual health services, seeking this information directly from the women themselves through discussion. The findings intend to highlight barriers and facilitators to services and encourage more comprehensive and inclusive approaches to HIV prevention.

This paper reports on qualitative research undertaken to identify factors that shape access to HIV and sexual health provision among women of African and Caribbean heritage in a local service in England. This was the first stage of a participatory health promotion project between Terrence Higgins Trust and African Families in the UK. Terrence Higgins Trust is the UK’s leading HIV and sexual health charity. The charity operates at both national and community level, prioritising the inclusion of people living with HIV across all levels of the organisation. African Families in the UK is a community interest company, which primarily exists to serve the interests of British African diaspora and other racially minoritised families. They offer training and support groups, as well as advocating for the rights of migrant communities by working with providers, including health care, to ensure their services are culturally appropriate and competent to meet the needs of different communities. The project was a response to the disproportionate impact of HIV among women of Black African ethnicity in England.^4^ This initial qualitative stage informed the development of health promotion videos, which aimed to raise awareness of HIV to reduce stigma and increase uptake in HIV testing among women of Black African and Caribbean heritage.

## Methods

### Study setting

This study was undertaken in a large council estate on the outskirts of a city in the south of England. According to the 2021 census, the population is 13,469, and it is an ethnically diverse area. African Families in the UK provide support in the area and had established a women’s group, through which they provided training and support sessions on topics such as health, education, financial management, domestic violence, as well as organising social activities and providing a food bank. These sessions took place in the local community centre where approximately 20 women would meet weekly. The group mostly consisted of migrant women aged between 25 and 50. The idea to conduct research and produce a new health promotion campaign to reach African and Caribbean women was formed during the meetings of this women’s group, in which a staff member from Terrence Higgins Trust (NJ) had delivered trainings on HIV and sexual health.

### Study design and participant sample

This study adopted a participatory action research framework, acknowledging lived experience as a basis of knowledge and centralising less heard from voices in the research outcomes.^15^ This paper reports on the findings from the qualitative data collection stage of the project.

Data were collected by three researchers (NJ, NJS, DR); all cis women, two of whom are of Black African and Black Caribbean heritage – therefore representing the research population. The third researcher is of white British heritage. NJS and DR are experienced community researchers and have previously conducted interviews and focus groups within their communities. DR and NJ both hold a PhD qualification and are experienced in conducting qualitative research. At the time of this study, DR and NJS were employed as consultants for African Families in the UK, and NJ was employed by Terrence Higgins Trust.

Purposive sampling was used to recruit adult women (aged 18+) of African and Caribbean heritage from the local area to focus groups or interview. The researchers attended the women’s group during one of their weekly meetings and informed everyone about the research, asking those who were interested in participating to contact one of the researchers. Most requested to join a focus group, and four women took part in a one-to-one interview. Whilst most of the participants knew each other, due to the flexible and informal nature of the women’s group, not all the women were familiar with one another. Although the data indicate HIV disproportionately affects Black African populations, this research explored how perceptions of HIV are shaped by social relationships. As such, the criteria for participation were broad, and the participants reflected the diversity and social dynamic of the existing women’s group, which was mostly made up of women of African and Caribbean heritage. Inclusion criteria were cis women over the age of 18 years. Participants were excluded if they were under the age of 18 years, resided outside of the county where this research was undertaken, or were not of African or Caribbean heritage.

### Data collection

This study was conducted between June 2021 and November 2021. Focus groups and interviews were undertaken in local community centres. Private rooms were used to ensure only the participants and researchers were present. Focus groups lasted approximately 2 to 3 hours, and interviews were conducted between 1 and 2 hours. These data were collected to gather insights to inform a health promotion project. Therefore, due to the aims and budget constraints, we did not set out to achieve saturation. However, similar patterns were beginning to emerge in the data. Field notes were taken during focus groups and interviews where possible, otherwise, they were recorded immediately afterwards.

All participants were provided with clear information about the study both in verbal and written form. This information included the purpose for conducting the study, an explanation for why they had been asked to participate, an overview of how the study would be undertaken, how the data would be used and stored, and the risks and benefits of participating. All participants were informed verbally and in writing that they were able to ask questions and request further information if needed, and informed they could withdraw from the study at any time without providing a reason. All participants signed a consent form.

The focus groups and interviews were audio recorded, transcribed verbatim (NJ), and de-identified. Once transcribed, all audio recordings were permanently deleted, and transcriptions were stored on a secure Google Drive.

Data were collected using focus groups and one-to-one interviews; the women chose their preferred method. Both the focus groups and interviews were semi-structured, using a topic guide to generate a relevant discussion.^16^ The guide included the talking points relating to HIV knowledge and awareness, culture, community, services, and perceptions of health promotion campaigns and materials. The guide was intentionally loose to allow for the emergence of unanticipated findings.^17^

The diversity of the data collection team allowed for both ‘insider’ facilitation, i.e. the researchers had a similar social and cultural background to the participants (NJS and DR), and ‘outsider’ facilitation, that is, the researcher had a different social and cultural background to the participants (NJ). This increased the strength of the study. Insider researchers are able to join social situations with pre-existing knowledge, which means they are less likely to experience culture shock, are better able to blend into existing social dynamics, and know how to approach individuals about culturally sensitive topics.^18^ However, they may also be perceived as too close to the participants’ social group. In contrast, outsider researchers are external to existing social relationships, and participants may be more willing to share personal information.^18^

The researchers intended to create an environment that would provide insight into the peer group interactions that produce social knowledge.^19^ Focus groups were used to study interactions among the women, enabling the researchers to understand how social dynamics shaped perceptions of HIV.^20,21^ Interviews were offered in recognition that some women may feel more confident to share their views one-to-one. Using different methods facilitated participation. Moreover, whilst focus groups are effective for encouraging conversation, the impact of social desirability bias may be more prevalent in a group setting.^22^ As such, in the interviews, the women tended to share more in-depth personal accounts of HIV.

The researchers did not conduct repeat interviews and focus groups due to the time constraints of the project. However, feedback sessions were held in which the researchers discussed the findings with participants to ensure fair and accurate representation.

### Ethics approval and consent to participate

Ethical approval was received from Oxford Brookes University Research Ethics Committee: UREC Registration Number: 211509.

All participants provided written consent to take part in the study.

### Data analysis

An applied thematic analysis was used to code data using deductive and inductive techniques.^23^ Deductive codes were based on the interview guide and existing literature, whilst inductive codes were added as data were analysed. Two of the researchers (NJ, SB) reviewed the transcripts manually and independently, identifying overarching themes, which form the structure of the findings. No data analysis software was used. NJ, SB, NJS and DR met regularly during the data analysis process to discuss thematic development. Crucial to this process were multiple meetings to discuss the themes with the community researchers, NJS and DR, to ensure fair interpretation of the results. This was a conscious decision to ensure that two White women were not solely analysing the experiences of Black women. The four researchers reached consensus on the following three key themes:

- Enduring fear of HIV rooted in past lived experiences
- Responsibility for HIV transmission and prevention
- Motivations to change attitudes and reduce stigma.

These themes were communicated to the research participants during in-person feedback sessions. The reporting of this study conforms to the COREQ statement.^24^

## Results

Five focus groups (see Table 1) and four one-to-one interviews were undertaken over a period of 6 months in 2021. In total, 23 women representing 14 different countries participated in this study (see Table 2). No participants requested to withdraw from the study. Participants were all aged between 18 and 65 years. One woman disclosed an HIV-positive status in a one-to-one interview. For all other women, their HIV status was disclosed as negative or undisclosed.

**Table 1.** Focus group participants.

Focus group	Number of participants
FG1	5
FG2	5
FG3	4
FG4	3
FG5	2
Total	19

**Table 2.** Participants’ country of origin.

Country of origin	Number of participants
Algeria	1
Democratic Republic of Congo	2
Dominican Republic	1
Egypt	1
Guinea	1
Jamaica	1
Kenya	5
Nigeria	3
Senegal	2
Sudan	1
The Gambia	2
Trinidad	1
United Kingdom	1
Zimbabwe	1
Total	23

### Enduring fear of HIV rooted in past lived experiences

Most participants were born and spent their formative years in countries that faced (and largely still are facing) a generalised HIV epidemic. As such, HIV had affected almost everyone in different ways, for example, loss of friends or family, experiences of caregiving, orphaned children, and being diagnosed with HIV. When asked about HIV, the women commonly reflected on memories of the AIDS epidemic in their countries of origin. These memories were shared through personal stories and vivid visual descriptions; one participant described a person they knew who had developed AIDS defining illnesses:

*‘. . . and there were boils all over his body. . . we started looking at him and wondering what is wrong with him, with all this* [sic] *boils all over his body?. . . our parents started telling us that this was a disease for people who are having sex anyhow.*’ (FG3)

The connection of these visual depictions of HIV with sexual behaviour, particularly the notion that people who contracted HIV were ‘*having sex anyhow*’, appeared to perpetuate a narrative of blame and a perception that HIV was brought in from outside:

‘*Everybody was angry that somebody went gallivanting and brought this back to the home, and now everyone was dying.*’ (FG3)

Others described a fear response when offered an HIV test:

‘*When I was asked to do the test, I was like “Oh my God, for what?*” *You know? Within me, I was praying*, “*God, I don’t want any problem*”.’ (FG4)

Some women displayed accurate knowledge about HIV treatment:

‘*There are some pills that can protect you to stay long. If you get infected you can still have a good, healthy life*’. (FG1)

Yet the trauma associated with HIV due to past experiences of loss and stigma has persisted, despite knowledge of effective treatment:

‘*But still, it’s depressing for people who have got the HIV because of the trauma. It’s traumatising.*’ (FG 3)

Trauma deriving from the loss of friends and family members as a result of HIV, fear, and stigma appeared to be a prominent factor in shaping people’s beliefs about HIV. One woman, who was living with HIV, explained how such beliefs had affected her mental well-being upon diagnosis:

‘*I was giving up. I was not opening my curtains or windows. I just told myself, I am done with life. . .. you can’t tell somebody that you have HIV in* [country of birth], *you can’t. It will be like a song* [gossip]. *What kills people now in Africa is stigma, and what people say*’. (Interview)

Misinformation, gossiping, and stigma undermine the advancements made in treating HIV, and often leave those who are diagnosed isolated from their communities. In this study, stories were often told in response to discussions about HIV treatment, directly challenging the idea that HIV is not something about which to be afraid. Fear of HIV was not only derived from a lack of knowledge about treatment and past experiences but also from misinformation, in which people living with HIV were portrayed as dangerous and manipulative, and wanting to intentionally infect others:

‘*Some people that I know, they have HIV, and they need to share it* [give it to others]*. . . They are wicked people*’. (FG1)

### Responsibility for HIV transmission and prevention

The likelihood of contracting HIV was discussed within the context of community, identity, and personal relationships. There was a relatively low perception of personal HIV risk among the women. Some women considered HIV to be an issue that affected people from other cultural or religious backgrounds, in which attitudes towards sex are more liberal. The responsibility for transmission was often perceived to be among western tourists, revealing a distrust of white western people travelling to African countries:

‘*. . .and you know some of these white people went to Africa, and they gave HIV to people there* [country of origin] *. . . yes, the tourists come like they’re trying to help us, but then they use our young girls and give them infections.*’ (FG1)

The idea that HIV infection is brought by foreigners or westerners travelling to African countries was consistent with some beliefs surrounding the origin of HIV:

‘*I heard that HIV, this disease, is man-made, and I heard that it was taken to Africa to test on Black people.*’ (FG2)

The exploitation of African people appears to have created a sense of distrust towards health care in England. Therefore, even where the perception of HIV risk was not low, barriers to services remained if the services offered reflected ‘white saviourism’:

‘*The cases are there, among Black women, but we don’t want a saviour, like a coloniser. . . It can feel like we are just the ones with all the problems, and they are our saviours*’. (FG1)

Others also acknowledged that they could be exposed to HIV infection but expressed that this was not related to their personal actions or behaviours, but rather the cultural norms or the norms within their relationships, which meant they had little agency over decisions surrounding sex and HIV prevention:

‘*The wife will be in the house, so the husband will go and do those things* [sex], *but because you are married it is so hard to say to him ‘will you go and have a test?’ To tell him that we have to go for a test every year, no I can’t do that*’. (FG4)

Whilst some women described challenges in negotiating safer sex with their partners, it is important to note that the women in this research were not passive victims. Some women expressed their agency by adopting other strategies, such as regular sexual health screens to test for HIV and STIs:

‘*My husband has a second wife, so when he goes there, by the time he is coming home, I just go and check myself, if I’m ok or if I need some antibiotics* [to treat STIs]’. (FG1)

Other strategies involved home treatments, which were shared via peer networks. Whilst there was a preference for more traditional, organic methods of infection control among some of the women, other home remedies involved the use of household chemicals, which they believed killed any infections, such as STIs and HIV, that may be present in semen:

‘*Dettol, it’s painful but it’s good. It kills all the infections. . . I also used to buy some gels and creams from Boots. . . but the Dettol is the best*’. (FG1)

Challenges in managing infection risk within their sexual relationships, combined with distrust in health services, may further motivate women of African and Caribbean heritage to use at-home treatments, such as vaginal douching with household detergents.

### Motivation to change attitudes and reduce stigma

Many of the women described HIV and sex as taboo topics that are not openly discussed:

‘*A lot of women wouldn’t want to sit here and talk about this HIV. A lot of women think it’s a taboo, I think this is the main reason why people shove this HIV under the carpet.*’ (FG2)

However, in recognition that the silence around HIV is a problem, most women who took part in this research showed strong motivations for change, particularly to protect younger generations:

‘*I want to suggest that, maybe we as parents, we can sit our kids down and be talking about these things. . . we can enlighten them and educate them.*’ (FG3)

For others, they were motivated to share the information they had learned about HIV testing and treatment to protect friends and family in their country of birth:

‘*See, for me, this* [HIV treatment] *is what I’m very very interested in. Especially the people back home, I want them to know this, and I want them to open up about it*’. (FG1)

Although some women stated that HIV and sex were taboo issues, many described positive experiences of the focus groups and were keen to continue the conversation, motivated to protect and mobilise others to break the stigma associated with HIV:

‘*I think we should do things like this more often to enlighten us, a lot of us are still living in darkness. And we need to break that stigma, we need to break it, we need to talk about everything, and we need to be there for each and every individual*’. (FG2)

The women showed appreciation for the group context and guided discussion about a sensitive subject, and valued what they had learned through the process:

‘*I want to say thank you to everybody in this group, it was nice to talk and hear all of you talking without any embarrassment. I have learned a lot about the medicine, and I am willing to do another one* [focus group] *if you do it*’. (FG2)

Many women expressed a personal responsibility for making community settings more inclusive for people living with HIV, as well as supporting them to access HIV prevention and treatment:

‘*But, now the people who have got this* [HIV], *how can we make them feel comfortable? They are stigmatised. We need to be having those conversations, and telling people that they can treat it, because it’s not like it used to be*’. (FG1)

Most women described sex and sexual health as taboo topics, however, there was a clear recognition that this was problematic as it discourages people from testing and isolates people living with HIV from their communities. In light of this, the women were motivated to find a solution.

## Discussion

This study sought to understand the social and cultural factors that shape perceptions of HIV and access to HIV prevention among women of African and Caribbean heritage. The findings provide contextual knowledge to national data showing that people of Black African ethnicity are disproportionately affected by HIV and late diagnosis,^4^ and local data showing that Black women are least likely to accept an HIV test when offered.^5^

The findings revealed how women of African and Caribbean heritage understand HIV through a myriad of personal experiences, trauma due to the loss of family and friends, early education about HIV during childhood, sharing misinformation or stories, and medical information. Such experiences have influenced perceptions of HIV transmission, responsibility for HIV prevention, trust in health care systems, and motivations to challenge HIV-related stigma. Three overarching themes arose from the findings.

### Fear, distrust, and stigma associated with HIV

In the UK, there is limited research on how trauma associated with HIV affects people who spent their formative years in a country facing a generalised HIV epidemic. Research has primarily focused on the trauma experienced by White gay and bisexual men in the 1980s.^25^ Whilst this is an important perspective, and one that is central to the UK’s history of HIV, it does little to represent the diverse experiences of the UK population.^26^ Moreover, the narrow focus on the AIDS crisis being in the 1980s and 1990s places trauma as something ‘historical’, overlooking people’s more recent experiences.^27^ This study has responded to this research gap and revealed important insights into how women of African and Caribbean heritage have understood and experienced HIV in the past, and how this shapes their engagement with prevention and treatment services.

Much of the literature on HIV acknowledges that trauma and fear hinder HIV care engagement and health-seeking behaviour.^28,29^ This study supports this claim, revealing how, when asked about HIV, the women most commonly recalled past memories associating HIV with illness and death. These memories were described with vivid visual descriptions of what AIDS ‘looks like’. The visual evidence of AIDS often represents people’s worst fears – helplessness in the face of a deadly virus – and thus HIV embodies feelings of terror and trauma.^27^ Trauma maintains a sense of fear associated with HIV, which can generate a feeling of ‘better off not knowing’, preventing the uptake of HIV testing.^30^

In response to the persisting fear, HIV and sexual health organisations have disseminated messages about U = U (undetectable = untransmittable) and ‘Can’t Pass It On’ to inform the public that people living with HIV, who are on effective treatment, can now live a long healthy life and cannot transmit the virus to others.^31,32^ However, many factors shape how this information is understood and accepted. Firstly, research shows that misinformation and conflicting accounts of health epidemics cause public confusion and create barriers to the acceptance of new information.^33^ Perhaps one of the biggest challenges for HIV campaigns to overcome is that ‘trusted organisations’, such as governmental and health care institutions, were the early sources of fear-based messaging about HIV.^34^ In this study, U = U contradicted the women’s personal experiences, which were articulated to push back against assertions that HIV is not something to be feared.

Another factor that shapes acceptance of the U = U message is how much the target audience engages with and trusts the source of the information.^35^ Through examining historical and present-day contexts, this research sheds further light on how engagement with HIV education is dependent on trust. In the UK, trust in health and social systems among African and Caribbean communities is shaped by colonisation and health inequalities.^36,37^ In the context of HIV prevention, distrust is compounded by ‘top-down’ health promotion initiatives, which ‘inform’ African communities that they have ‘problems’, which can be solved by the organisation’s ‘solutions’ – solutions that predominantly focus on behaviour change.^38^ Some women likened this approach to white saviourism and felt suspicious of individuals of white ethnicity disseminating messages about HIV within their social spaces. Moreover, top-down health promotion places the obligation for change on the individual, simultaneously absolving health systems of their responsibility for health inequalities and HIV disparities.^39^ Some women rejected this approach and showed a preference for sharing information and learning through peer networks. Peer networks appeared to include friends and family – particularly intergenerationally from mothers and grandmothers. Traditional healers, a recognised practice in some African countries, were not mentioned. Some women did not agree with home remedies, whereas others preferred to use home remedies. Overall, there was an acknowledgement that if home remedies don’t work then go to the doctors. It appeared that the women were not solely accessing peer networks but for many this was the first port of call.

Whilst some women accepted the U = U message they continued to push back against the notion that HIV was not something to fear. Misinformation about intentional HIV transmission was shared in response to assertions that HIV had changed. According to Heller [35], people perpetuate rumours to resist information that is provided by institutions that are not trusted. This misinformation is particularly problematic as it reinforces stigmatising narratives of people living with HIV as dangerous and infectious.^40^ Moreover, when misinformation connects with and confirms past lived experiences, it is more likely to be accepted and remembered than a medically complex message, such as U = U.^41^ As such, the provision of information about HIV treatment alone is not enough to reduce fear, stigma, and encourage uptake of HIV testing. Organisations must recognise peer networks as important avenues for information sharing, and where possible, work with these networks to raise awareness of HIV over time.

### HIV as a taboo issue and perceptions of risk

This study revealed that motivations to test for HIV and take up HIV prevention methods were influenced by perceptions of HIV risk, agency in reducing the risk of HIV acquisition, stigmatising beliefs, and HIV as a taboo issue, which encouraged some to distance themselves from the topic.

Perpetuating misinformation may enable people to distance themselves from stigmatised attributes, such as HIV, by associating it with others who do not belong to their social group.^42^ In this study, some women dissociated themselves from HIV by suggesting that tourists and people who ‘have sex anyhow’ are responsible for HIV transmission. In turn, this lowered their perception of their likelihood of contracting HIV. This is an important finding as the acknowledgement of HIV infection risk is associated with the uptake of prevention methods.^43^ However, Kaufman et al.^44^ suggest that risk awareness campaigns must be used with caution as they may inadvertently exacerbate stigma, framing HIV as a marker of risk-taking and irresponsibility.^45^ Exacerbating stigma may further motivate people to distance themselves from HIV, resulting in a lower uptake of testing.

Risk awareness campaigns are also unlikely to be effective if the target audience lack agency to reduce their risk of contracting HIV. Some women in this study acknowledged their potential risk of acquiring HIV, but felt that it was not a result of their behaviour but rather that of their male partners. Many women described difficulties in openly discussing HIV and sexual health with their partners because it is considered ‘taboo’. PrEP may be particularly useful for women who face challenges in negotiating condom use with their partners. However, Nakasone et al.^46^ found that women of Black African and Black Caribbean heritage acknowledge the effectiveness of PrEP but prefer prevention tools that will facilitate open discussions with their partners, building trust and intimacy and encouraging joint responsibility for HIV prevention. In contrast, PrEP is perceived to be a tool that would circumvent those discussions. Promoting HIV testing and/or PrEP within the context of a woman’s relationship with her partner, and focusing on its benefits for trust and intimacy, may be more successful in motivating women to take up HIV prevention methods.

Despite awareness of the availability of HIV and STI testing and treatment, some women expressed preferences for home remedies, such as washing with Dettol, which were shared through social networks. If health institutions are not a trusted source of information, people are more likely to rely on information from their peers.^46^ Whilst peer networks are key for raising awareness of HIV and encouraging others to test, they can be problematic if stigmatising narratives and harmful practices that comprise women’s health are shared.

Furthermore, whilst peer networks are often relied upon for information about health care, including sexual health and contraception, HIV is less talked about within these networks.^46^ The women in this study described HIV as something that has been ‘shoved under the carpet’ because people consider it to be taboo. This means that HIV prevention campaigns within African and Caribbean communities are unlikely to see the same success as with White gay and bisexual men, whereby networks of sexual health information are often established and HIV testing is normalised. The ‘taboo’ prevents open discussions and maintains stigma associated with HIV.

### Value of learning in a safe group context

In this study, women discussed HIV within a focus group setting, which is a useful tool for gaining insight into social dynamics and peer relationships.^19,21^ There is also evidence that focus groups can foster a sense of empowerment and resilience, motivating people to take action.^15^ In this study, the researchers left the direction of the discussion open to the women participating, only using the topic guide when needed, for example, the conversation moved on to topics unrelated to the aims of the research. This facilitated a free and open conversation. Consequently, the focus groups in themselves became a safe environment for learning; the women opened up about HIV, asked questions, and shared stories and memories. They were also open to learning new information when the facilitators gently challenged misinformation. In turn, as the focus group discussions progressed, the women became increasingly motivated to change the narrative about HIV within their communities.

Similar to findings from Opara et al.,^15^ this study revealed clear and strong motivations among women of African and Caribbean heritage to raise awareness of HIV and reduce stigma. This motivation is not well documented in existing literature, which is often primarily concerned with HIV risk, stigma, and barriers to HIV prevention, as opposed to strength, resilience, and motivation for change.^43,47,48^ This has resulted in an over-reliance on top-down health promotion efforts whereby HIV education is provided, often by an outsider, as a method of inducing behaviour change. This research has highlighted the value of peer learning, which allows people to explore sensitive topics thoroughly and without judgement. This approach is likely to be more effective than primarily focusing on disseminating information about HIV testing and U = U in isolation of local cultural and social contexts.

### Limitations

There are some limitations of this study. First, the interview guide was not formally pilot tested, in line with the pragmatic aspects of this study. However, the guide was reviewed by the research team and a community leader outside of the research team prior to data collection, and as such did go out to consultation. Second, the sample size is small and limited to one geographical area in the south of England so the findings may not be representative of women of African and Caribbean heritage across the UK. However, that was not the intention of this research, which set out to understand and respond to local HIV testing trends. Moreover, this paper does not claim the findings are generalisable but argues the importance of implementing localised HIV prevention interventions alongside national programmes.

## Conclusion

Multiple factors shape the understanding of HIV and uptake of HIV prevention among women of African and Caribbean heritage. Local, community-specific, and participatory HIV prevention interventions are needed to understand and respond to these factors. Tailored rather than targeted interventions can better respond to trauma and fear, effectively build trust, and facilitate stigma reduction. Encouraging open discussion through focus groups and interviews prior to designing local campaigns effectively includes community members from the outset. Such approaches will complement national campaigns, such as ‘I Test’, and continue to destigmatise and normalise HIV testing across different communities.

## Supplemental Material

sj-pdf-1-whe-10.1177_17455057251385798 – Supplemental material for Breaking barriers: A qualitative study exploring the social and cultural factors that influence HIV awareness and uptake of HIV prevention among women of African and Caribbean heritage in England Supplemental material, sj-pdf-1-whe-10.1177_17455057251385798 for Breaking barriers: A qualitative study exploring the social and cultural factors that influence HIV awareness and uptake of HIV prevention among women of African and Caribbean heritage in England by Nicola Jones, Sarah Bekaert, Dianne Regisford and Nicole Jameelah Shodunke in Women's Health
